# Editorial: Anti-transgender prejudice: causes, consequences, and interventions

**DOI:** 10.3389/fpsyg.2023.1282109

**Published:** 2023-09-12

**Authors:** Thomas J. Billard, Jeffrey H. D. Cornelius-White, Naiara Ozamiz Etxebarria

**Affiliations:** ^1^School of Communication, Northwestern University, Evanston, IL, United States; ^2^Center for Applied Transgender Studies, Chicago, IL, United States; ^3^School of Mental Health and Behavioral Sciences, Missouri State University, Springfield, MO, United States; ^4^Evolutionary Psychology and Education, University of the Basque Country, Bilbao, Spain

**Keywords:** transphobia, anti-transgender prejudice, voice type, stigmatized identities, trans youth, antidiscrimination education, transgender and gender diverse

Public attention to transgender people has increased exponentially in recent decades around the world. Transgender visibility in media and culture has made the populations of many countries more aware (if not more understanding) of trans identities (Billard and Nesfield, 2021). At the same time, the very fact of trans people's existence has been made an issue of political controversy by global far-right movements and, counterintuitively, certain segments of self-proclaimed feminist movements (Bassi and LaFleur, 2022; Billard, 2023, 2024). As a result, the variety and scale of the problems trans people encounter in daily life has increased—problems in urgent need of solutions.

Scholarly attention to transgender people has also increased. However, within the social sciences, studies of trans people have been fragmented—archipelagated across various academic disciplines without dialogue among them—and often motivated by the “novelty” of transgender topics, taking a “current affairs” framing of their significance (Billard, 2019; Billard et al., 2022). Within the humanities, the nascent field of transgender studies has emerged as a robust area of scholarship, but with a firm rooting in critical theoretical and cultural studies traditions and an ideological aversion to empiricism that has prevented the field from addressing the material realities facing trans communities (Billard et al., 2022; Amin, 2023). Meanwhile, medical research on trans people has struggled with a long history of pathologization and a sometimes-problematic funding link between transgender medicine and HIV/AIDS research (Kanamori and Cornelius-White, 2016; Aizura et al., 2020).

Recognizing both the urgency of the problems facing trans people and the need for scholars to mobilize the tools of research to address those problems, some within transgender studies have called for a reorientation of the field toward an “applied transgender studies” (Johnson et al., 2021; Billard et al., 2022; Felt et al., 2022; Hoffmann, 2022; Johnson, 2022). As Felt et al. (2022) summarize this emergent approach, applied transgender studies “merges critical traditions of trans studies with technical and methodological contributions from the social sciences to facilitate work which can improve the material conditions of transgender life” (p. 44). The Center for Applied Transgender Studies in Chicago and its flagship journal, the *Bulletin of Applied Transgender Studies*, currently serve as venues for advancing this applied vision, but the present Research Topic answers Billard et al. (2022) call for scholars of transgender topics more broadly to join in these efforts.

While the scholarly literature on prejudice against transgender people is robust (e.g., Kanamori and Cornelius-White, 2016, 2017; Kanamori et al., 2017, 2023; Billard, 2018; Ozamiz-Etxebarria et al., 2020), and some has focused on identifying solutions (Gorrotxategi et al., 2020; Smith Nelson and Cornelius-White, 2022), much has not. Thus, we intentionally focused this Research Topic not merely on the “causes” and “consequences” of prejudice, but also on “interventions” to address it. Moreover, we aimed to disentangle the various social and personality dynamics involved in anti-transgender prejudice in a manner that attends to how those dynamics differ across various national and cultural contexts, so as to address the needs of *all* trans people.

The four articles included in this Research Topic represent a variety of theoretical and methodological approaches to the applied study of anti-transgender prejudice. In the only published article from the Research Topic explicitly focused on interventions, Flores et al. showed that antidiscrimination exercises could help individuals exposed to political advertisements about transgender rights develop less exclusionary attitudes across two survey experiments conducted in the United States. However, of two different antidiscrimination exercises—describing a personal narrative of discrimination and perspective-taking—only one—perspective-taking—was successful in decreasing exclusionary attitudes. Moreover, they found evidence of potential “backfire” effects, particularly among participants who refused to participate in the perspective-taking exercise.

In another article based on two studies conducted in the United States, Totton et al. investigated the role of intentionally and unintentionally disclosing a stigmatized identity in individuals' prejudice toward people with that identity. Comparing the disclosures of atheist and transgender identities, they found that the disclosure of transgender identity resulted in greater levels of prejudice and stronger perceptions of deceptiveness. This effect was further amplified when the disclosure of identity was unintentional, rather than intentional, with perceived deception mediating the relationship between disclosure type (intentional or unintentional) and levels of prejudice.

In a study concerned with visual bias in voice type appraisal, Knight et al. compared the performance of cisgender and transgender participants in distinguishing six voice types (e.g., soprano, bass, etc.). Participants were asked to appraise voice type with audio only, video only, or combined audio-video to assess the degree to which visual cues biased appraisals. Knight et al. found large visual bias across the full range of voice types. Roughly a third of the distance across the appraisal of each voice range (e.g., soprano) was attributed to visual bias. However, this bias was significantly smaller amongst transgender participants than cisgender participants. In other words, transgender participants were able to more accurately assess voice type with less visual bias.

The final study of the Research Topic narrows in on the family, investigating the role of parents' prejudice against their transgender children in those children's experiences of child abuse and subsequent non-suicidal self-injury. Drawing on a survey of trans men and women in the Guangdong province of China, Cao et al. found that trans people's experiences of childhood abuse was associated with greater emotional dysregulation and more self-harm. These findings not only point to the need to address root causes of prejudice to minimize trans youths' experiences of abuse, but also point clinicians to important intervention points for supporting trans people with experiences of abuse in their childhood.

Taken together, the articles included in this Research Topic represent a modest contribution to the collective effort of solving the problem of global anti-transgender prejudice. And prejudice is only one of countless issues facing trans people in need of urgent solutions. We hope these works inspire further scholarship that similarly aims to address the dire realities facing trans communities globally and advances recent developments within the academy that take researchers' responsibilities to trans communities more seriously.

## Author contributions

TB: Writing—original draft, Writing—review and editing. JC-W: Writing—original draft, Writing—review and editing. NO: Writing—review and editing.
